# Postpartum women’s perception of stressors in the delivery ward: a qualitative study

**DOI:** 10.1186/s13104-020-05176-1

**Published:** 2020-07-13

**Authors:** Farideh Kazemi, Seyedeh Zahra Masoumi, Farzaneh Soltani, Khodayar Oshvandi, Samereh Ghelichkhani, Ziba Niazy

**Affiliations:** Department of Midwifery, Mother and Child Care Research Center, School of Nursing and Midwifery, Hamadan University of Medical Sciences, Hamadan, Iran

**Keywords:** Postpartum period, Stressors, Vaginal delivery, Qualitative research

## Abstract

**Objective:**

Delivery is a challenging event in women’s lives. What happens during childbirth is stressful for most women. Regarding the short and long-term effects of stressors of the delivery ward on childbirth and neonatal outcomes, and given that understanding the stressors are influenced by existing social culture and factors, the present qualitative study was conducted to investigate women’s perception of stressors in the delivery ward. Participants were 13 newly delivered women who were monitored in the recovery room at the Fatemieh Hospital of Hamadan, Iran. Purposive sampling was performed and continued until data saturation. The data collection method was in-depth and semi-structured.

**Results:**

The content analysis of qualitative data led to the emergence of two themes of human stressors and environmental stressors as well as four categories; i.e., personal factors, care staff-related factors, environmental factors, and equipment-related factors. The research findings indicated that several personal, environmental, staff-related, and equipment-related factors could lead to stress in the labor and delivery ward. Although ignoring them and increasing stress during childbirth may jeopardize the childbirth consequences, most of them are ignored and overlooked. It is necessary to consider measures to control these factors as much as possible.

## Introduction

Stress is a reaction to a perceived real or imagined threat [1]. Pregnancy, in addition to being a period of happiness, also causes high physical and mental stress for the mother and can be associated with various psychological problems [2, 3]. The transition to motherhood is an important part of life and increases a mother’s vulnerability to depression, anxiety, and stress. Prenatal psychiatric problems are among the most important public health issues. High levels of such stresses during pregnancy are associated with adverse psychological and physiological consequences for infants and mothers [4]. The prevalence of pregnancy anxiety in different parts of the world has been reported to be 14–54% [2]. They suffer from high levels of fear of childbirth [5]. Pregnant women are vulnerable to stress and high levels may increase the risk of prenatal anxiety and depression [6]. Moreover, it is an important risk factor for pregnancy complications [7]. The importance of this issue is even more highlighted regarding that exposure to stressful environments can be associated with poor outcomes [8]. Although the experience of childbirth is an unpredictable phenomenon, it should be a positive memory with minimal risk to the mother [9]. About 7–35% of women report a negative experience of childbirth [10]. Several factors affect the experience of mothers after childbirth, including individual social characteristics, social status, expectations of the mother, prenatal education, maternal awareness, pain perception, type of delivery, instrumental delivery, unforeseen complications, medical interventions, transfer of the infant to the neonatal intensive care unit, and adequate support during labor and delivery by the medical staff [11, 12]. When childbirth is difficult and stressful, most mothers experience a feeling of indifference and apathy toward the baby in the 1st days after delivery. This feeling disrupts the warm and intimate relationship between mother and child such that an insecure relationship is formed between mother and child [13, 14]. Moreover, anxiety and stress associated with pregnancy can trigger psychological and mood disorders in the mother and her child in the future. Women who have a negative experience of childbirth remember the birth of a child as a process of grief, sadness, pain and anger, and suffer from complications such as postpartum depression, fear of childbirth, post-traumatic stress disorder, unsuccessful breastfeeding, and increased miscarriage in subsequent pregnancies. Also, in future pregnancies, the preference for selective cesarean section increases [10, 15]. Given the short-term and long-term effects that stressors can have on labor and neonatal outcomes, and given that perceptions of stressors are influenced by community culture and social factors, the present qualitative study was conducted to examine women’s perceptions of the childbirth stressors.

## Main text

### Methods

The present study is a qualitative research conducted with a conventional content analysis approach. The research population consisted of all newly delivered women who were in the care unit of Fatemieh Hospital of Hamadan.

#### Inclusion criteria

The inclusion criteria of this research include being primigravida women, singleton fetus, hospitalization at week 37 and above, and lack of chronic medical diseases or pregnancy complications. If the mother refused to continue during the interview, she would be excluded.

#### The data collection tools

The tool used in this study was a semi-structured and researcher-made questionnaire. The interviews were continued with the same questions after several pilot interviews and confirmation by the research team.

#### Research question

The main interview questions were as follows: How did you feel when entering the labor ward? How was your time in the maternity ward? What were the factors in your delivery that made you stressed?

During the interview, whenever the researcher felt that there was a need for a deeper understanding of the issue, questions were asked to elaborate.

#### Setting and recruitment

In the present study, purposive sampling was performed with maximum variation in maternal age, education, and social class and continued until data saturation. Next, 13 mothers were interviewed after the childbirth.

#### Data collection

The women were first given explanations about the study and the inclusion criteria were checked. Then, the individual interviews were conducted in the case of their consent. The interview lasted from 15 to 30 min. Interviewees’ voices were digitally recorded in the case of their consent and then transcribed by the researcher after the completion.

#### Analysis

Four criteria presented by Guba and Lincoln, including Credibility, Conformability, Dependability, and Transferability, were used to examine the accuracy of data in the present study [16].

- To gain credibility, we used long working on the subject, dedicated time to data collection, member matching, external check (investigating codes and classes extracted from interview texts by qualitative research professors), and integration in selecting participants and searching for the conflicting evidence.
- To increase the conformability, the researcher abandoned the assumptions and thoughts, and carefully documented all stages of the research, including data collection, analysis, and formulation of variables so that the external auditor could review the performance of the task and codes.

The following methods were applied to increase the dependability of data:

1. External observers were asked for assistance in addition to the research team members. To this end, the interviews were encoded independently by two reproductive health professionals to identify discrepancies in coding. Suggestions were provided in the subsequent interviews.

2. The code-recode method was also used during the data analysis. For this purpose, a part of the data was encoded and the same data were re-coded after at least two weeks. Eventually, the results of two codings were compared and the data reliability was determined by obtaining common codes.

- In the present study, rich, detailed, and step-by-step descriptions were used to enhance the data transferability. Every step of the study was written such that to allow the follow up of the path for others. Besides, it was attempted to select the most appropriate and most varied samples. MAXQDA-12 was used to analyze data.

### Results

In the present study, 13 pregnant women at an age range of 18–39 years and gestational age of 37–41 weeks were interviewed.

Data analysis began with the interviews. A total of 126 primary codes were extracted as a result of the initial encoding, and then the main codes were named based on similarities between the initial codes. At this stage, 34 main codes and 12 subcategories were obtained. The integration and classification processes continued and the main categories were created. Finally, the qualitative findings were summarized in 4 categories and 2 themes (Table 1).

**Table 1:** Themes, categories, and subcategories extracted from the study

Theme	Category	Subcategory
Human stressors	Personal factors	Hearing the Troubles
		Fear of therapies
		Fear of harm to self-health
		Fear of fetal health
	Care staff-related factors	The presence of a large number of medical staff
		Misbehavior of care staff
		Not paying attention to the patient’s status
Environmental stressors	Environmental factors	Noise
		Improper labor space
	Equipment-related factors	Lack of equipment and facilities
		Fear of hospital equipment

The “human stressors” theme consists of 2 categories, namely personal and care staff-related factors.

The “personal factors” category consists of 5 subcategories: (1) Hearing troubles, (2) Fear of therapies, (3) Fear of childbirth, (4) Fear of harm to self-health, and (5) Fear of fetal health.

In the “Hearing troubles” subcategory, listening to the experiences or beliefs of those around mother and her friends was described as stressors in the labor and delivery room:

Participant 1: “My sister-in-law said that their neighbors were there at the time of giving birth. They shut at me, hit my foot, and said why I got pregnant. So, I had stressed to receive any shut since the beginning of the pregnancy.

Participant 3: “Doctors come at my bedside and talk about horrible accidents they have experienced. This increases my pain and doubles the stress”.

In the “Fear of therapies” subcategory, some participants found treatment procedures stressful:

Participant 2: “They bothered me for examination and forced to have an examination, but I did not want to be examined by numerous individuals”.

In the “Fear of delivery” subcategory, some participants described childbirth as a frightening process and cited reasons for their fear:

Participant 11: “I was scared of its pain as it was my first experience”.

Participant 8: “I was afraid if I wouldn’t be able to give birth”.

In the “fear of harm to self-health” subcategory, some participants feared that their health would be compromised during the childbirth:

Participant 6: “I was scared that my pain wouldn’t start and I must go for the cesarean section, and people said the cesarean caused much blood loss”.

In the “fear of fetal health” subcategory, the concern about fetal health was a case that was almost mentioned by all mothers.

Participant 11: “I was always afraid if my baby had strabismus or less than five fingers”.

Participant 7: “I was always afraid if my baby had strabismus or less than 5 fingers”.

The care staff-related category consisted of three sub-categories: (1) High attendance of care staff, (2) Misbehavior of care staff, and (3) Not paying attention to the patient’s status.

In the “high attendance of care staff” subcategory, the frequent attendance of care staff and crowded ward were stressors of the delivery ward:

Participant 1: “The noise, crowded ward, and a lot of individuals in green and white clothing were all stressful”.

Participant 8: “There were a lot of nurses and they were continuously commuting, so it made me stressed”.

In the “misbehavior of care staff” subcategory, the misbehavior of some staff, not giving information to mothers, and their misconduct caused stress in the labor and delivery wards.

Participant 8: “They were arguing with each other. I was scared to be distracted during the examination and my baby would die and they would not understand”.

Participant 4: “They were rampaging, so I was stressed”.

In the “not paying attention to the patient’s status” subcategory, the mother’s inappropriate situation in labor, and lack of privacy were the sources of fear:

Participant 3: “My feet could not be fixed on the labor bed; my serum was not attached and was fallen out; and my stress became doubled”.

Participant 10: “Sometimes, there were curtains, but its clamps were removed and the patient in the front bedroom could see me”.

The “environmental stressors” theme also consists of two categories, environmental and equipment-related factors. The “environmental factors” category consists of two sub-categories: (1) Noise and (2) Inappropriate laboratory space.

In the noise subcategory, the ward noise was a factor that was mentioned by almost all participants:

Participant 11: “I had greater pain and was scared when I heard the other women scream”.

Participant 9: “Here, we are afraid of the next bed; it is not good at all to be in a crowded room, and it gives a sense of fear”.

The “equipment-related factors” category also consists of two subcategories: (1) Lack of equipment and facilities and (2) Fear of hospital equipment.

In the “lack of equipment and facilities” subcategory, the equipment failure and lack of equipment and facilities caused fear in some mothers:

Participant 6: “There was a device that was listening to my heart, and it seems that its wires were broken and cut off. I thought my baby’s heart was in trouble.”

Participant 10: “They should increase the equipment to reduce our concern. As far as I see, there is just one from everything in the ward, for example, there should be enough number of the birth balls”.

In the “fear of hospital equipment” subcategory, it was stressful for some participants to see devices and equipment:

Participant 13: “How can I say; I’d be scared when I hear the name of that hospital”.

Participant 7: “I am partially scared of hospital equipment. I wish I was giving birth to my house, and someone helped me do it”.

### Discussion

The outcomes of the present study resulted in 2 themes of human and environmental stressors and four categories; namely, personal factors, care staff-related factors, environmental factors, and equipment-related factors.

#### Personal factors

Hearing the troubles, Fear of therapies, Fear of delivery, Fear of harm to self-health, and Fear of fetal health are the subcategories of personal factors. A qualitative study on primigravida women in Turkey indicated that childbirth pain was a cause of women’s delivery of fear. Moreover, it was reported that it included factors such as the inability to give birth and fear of labor pain and dying during childbirth. Some participants were also afraid of problems and procedures used during childbirth, such as episiotomy, vaginal examination, and the use of forceps or vacuum. Hearing negative stories about childbirth and health care personnel from friends and family, receiving negative information from the media, and seeing negative images of childbirth in the media were also considered as fear-related factors [17].

In studies performed during pregnancy and in people who have not yet experienced childbirth, fears of an episiotomy were more important to them [18]. A study of pregnant women in Australia revealed that lack of self-confidence at birth, fear of the unknown, internalization of other women’s negative stories about tear of the perineum, and labor pains were common concerns for primigravida mothers [19].

#### Care staff-related factors

The unnecessary presence of the care staff, misconduct of care staff, and the lack of attention to the patient’s status, and mother’s ignorance of the processes ahead were other stressors that were mentioned by mothers in the present study. Delaram et al. also mentioned congestion as a stressor in the delivery room [20]. Inattention to the mother’s status leads to higher fear and stress in her and causes concerns about harm to herself or her fetus [21]. In a study aimed at determining the social and personal factors related to the experience of childbirth in mothers, it was reported that the support of obstetricians and midwives in the labor and delivery ward played an important role in satisfying normal childbirth [22].

#### Environmental factors

Fear of hospital space, noise and inappropriate labor environment and from other patients, overcrowding of staff and treatment personnel, and lack of enough space in the labor were stressful environmental factors that were mentioned by mothers in the present study. In a study by Delaram et al., being exposed to unfamiliar environments, women scream during the delivery, inappropriate odor in the room, cold and warm room, dirty room, wall and door colors, and low or high light were among stressful environmental factors [20].

#### Equipment-related factors

In the present study, women talked about the lack of equipment and facilities and the high number of unnecessary equipment in the delivery and labor ward that worries pregnant mothers. Besides, some women stated that too much equipment in the ward led to their stress. Delaram et al. found that different apparatus and bed failures were stressors in women admitted to the labor and delivery ward [20]; these results are consistent with the findings of the present study. Delaram et al. found that women’s exposure to unfamiliar environments and the screaming of women giving birth were the most stressful environmental factors. Furthermore, performing vaginal examinations, specific labor status, abdominal pressure to accelerate labor, perineal incision, repair, and repeated student examinations were stressors reported by mothers [20].

## Limitation

One of the limitations of our study was that the interviews were conducted in public rooms for postpartum women due to the lack of a private room for them. Another limitation was the insufficient number of participants involved in this research to conclude. Therefore, the research process is continuing and future results will be added to these preliminary stages.

## Data Availability

The data used during this study are available from the corresponding author on reasonable request.

## Abbreviation

MAXQDA: MAX Qualitative Data Analysis
